# Clinical Value of ctDNA in Hematological Malignancies (Lymphomas, Multiple Myeloma, Myelodysplastic Syndrome, and Leukemia): A Meta-Analysis

**DOI:** 10.3389/fonc.2021.632910

**Published:** 2021-03-04

**Authors:** Xiangyu Tan, Han Yan, Lei Chen, Yuyang Zhang, Chunyan Sun

**Affiliations:** ^1^Department of Hematology, Union Hospital, Tongji Medical College, Huazhong University of Science and Technology, Wuhan, China; ^2^Collaborative Innovation Center of Hematology, Huazhong University of Science and Technology, Wuhan, China

**Keywords:** circulating tumor DNA, hematological malignancies, lymphomas, multiple myeloma, myelodysplastic syndrome, leukemia, meta-analysis

## Abstract

**Background:** Circulating tumor DNA (ctDNA) has offered a minimally invasive approach for the detection and measurement of cancer. However, its diagnostic and prognostic value in hematological malignancies remains unclear.

**Materials and methods:** Pubmed, Embase, and Cochrane Library were searched for relating literature. Diagnostic accuracy variables and disease progression prediction data were pooled by the Meta-Disc version 1.4 software. Review Manager version 5.4 software was applied for prognostic data analysis.

**Results:** A total of 11 studies met our inclusion criteria. In terms of diagnosis, the pooled sensitivity and specificity were 0.51 (95% confidence intervals (CI) 0.38–0.64) and 0.96 (95% CI 0.88–1.00), respectively. The AUSROC (area under the SROC) curve was 0.89 (95%CI 0.75–1.03). When it comes to the prediction of disease progression, the overall sensitivity and specificity was 0.83 (95% CI 0.67–0.94) and 0.98 (95% CI 0.93–1.00), respectively. Moreover, a significant association also existed between the presence of ctDNA and worse progression-free survival (HR 2.63, 95% CI 1.27–5.43, *p* = 0.009), as well as overall survival (HR 2.92, 95% CI 1.53–5.57, *p* = 0.001).

**Conclusions:** The use of ctDNA in clinical practice for hematological malignancies is promising, as it may not only contribute to diagnosis, but could also predict the prognosis of patients so as to guide treatment. In the future, more studies are needed to realize the standardization of sequencing techniques and improve the detection sensitivity of exploration methods.

## Introduction

In recent years, significant progress in the diagnosis and treatment of hematological malignancies has been made. However, the current gold standard for disease diagnosis and monitoring, tissue or bone marrow (BM) biopsy, is invasive and painful. A further issue is that sampling a single tumor site during biopsy may not reveal all malignant clones (1).

Mandel and Metais first described the presence of DNA molecules in human plasma in 1948 (2). In 1994, Vasioukhin et al. described the presence of tumor-specific mutations in cell-free DNA (cfDNA) of patients with myelodysplastic syndrome (MDS) and acute myeloid leukemia (AML), demonstrating the significance of circulating tumor DNA (ctDNA) analysis in hematologic malignancies (3, 4). Unfortunately, due to the lack of sensitive and specific detection methods, the research on cfDNA is relatively behind. With the recent advent of new techniques, such as droplet digital polymerase chain reaction (ddPCR) and next-generation sequencing (NGS), tumor-derived fragmented DNA in the plasma or serum, known as ctDNA, has the ability to be one of the most sensitive, non-invasive biomarkers available for use in cancer patients (5). Compared with a classic biopsy, ctDNA is more convenient and presents minor procedural risk to the patient, with a less expensive price. And more importantly, ctDNA has been determined to have a half-time of 16 min to 2.5 h in circulation, which enables ctDNA analysis to be considered as a real-time snapshot of disease burden (6). In theory, ctDNA could also deliver more complete information regarding the patient's entire tumor burden, because the sample may represent all tumor DNA present in the circulation, without spatial limitations of the biopsy sampling of a single lesion within a single anatomic site (7).

Although many recent studies have focused on applications in hematological malignancies (8–13), the results are still unclear. Therefore, we performed a meta-analysis to estimate the clinical application value in patients with hematological malignancies.

## Methods

### Search Strategy

We searched for studies in Pubmed, Embase, and Cochrane library with no restriction of publication date using key words “lymphomas AND ctDNA,” “myeloma AND ctDNA,” “myelodysplastic syndrome AND ctDNA,” and “leukemia AND ctDNA.”

### Inclusion Criteria, Exclusion Criteria

Two reviewers evaluated potential articles independently, according to the inclusion and exclusion criteria mentioned below. Discrepancies were resolved by discussion until a consensus was reached.

Inclusion criteria: (1) retrospective and prospective observational cohort studies involving patients with lymphoma, multiple myeloma, myelodysplastic syndrome, or leukemia; (2) ctDNA was analyzed in patients; (3) information on the diagnostic, prognostic, or predictive value of ctDNA was provided; (4) prognostic studies had to report the results of a survival analysis in the form of a hazard ratio and 95 % confidence intervals; (5) a sample size≥5; (6) reported in English; and (7) participants were adults.

Exclusion criteria: (1) cell-free DNA without information of mutations; (2) circulating viral DNA; (3) lack of outcomes; and (4) conference abstracts, comments, reviews, case reports, or meta-analyses.

### Data Extraction

Only full-text articles were taken into consideration. Extracted study characteristics included: first author, publication year, type of cancer, number of patients, ctDNA measurement method, and mutation evaluated in ctDNA. If the eligible studies provided survival data, hazard ration (HR) for overall survival (OS), progress-free survival (PFS), event-free survival (EFS), or disease progressing with 95% confidence interval (CI) they were extracted.

### Studies' Quality Assessment

The results of diagnosis quality assessment were shown in Table 1. The quality of prognostic studies was assessed by an adapted version of the reporting recommendations for tumor marker prognostic studies (REMARK) criteria for biomarker studies (Table 2) (14). Detailed information on quality assessment about prognostic studies was shown in Table 3.

**Table 1:** Results of diagnosis quality assessment of included studies according to the QUADAS-2 tool criteria.

**Study**	**Risk of bias**				**Applicability concerns**		
	**Patients selection**	**Index text**	**Reference standard**	**Flow and time**	**Patients selection**	**Index text**	**Reference standard**
Fontanilles, M.2017	L	L	L	L	L	L	L
Hickmann, A.K.2019	U	L	U	L	L	L	L
Mazzotti.C 2018	U	L	L	L	U	L	L
Sakata-Yanagimoto, M 2017	U	L	L	U	L	L	L
Watanabe, J. 2019 PCNSL	L	L	L	L	L	L	L

*L: low risk of bias; H: high risk of bias; U: unclear risk of bias*.

**Table 2:** A study could be allocated one point for each of the seven criteria; in case of ambiguity, half a point was assigned.

**Adapted REMARK criteria for quality assessment (1 point/criteria)**	
**1**.	Case selection adequate (baselines from medical chart)
**2**.	States the marker examined and the aim of the study
**3**.	Reporting at least the following characteristics: disease stage, histology, and received treatment
**4**.	States the time and type of sampling (serum/plasma)
**5**.	States the assay methods used and provides a detailed protocol (at least cfDNA isolation, sequence method, and sequence depth)
**6**.	A clear description of the flow of patients through the study
**7**.	A clear description of the reasons for dropout

*A study was included for further analysis when graded ≥ 5.5 points*.

**Table 3:** Prognostic studies were scored according to the criteria in Table 2.

**Study**	**1**	**2**	**3**	**4**	**5**	**6**	**7**	**Total**
Roschewski, M.2015	1	1	1	1	1	1	1	7
Assouline, S. E.2016	1	1	1	1	1	1	1	7
Kurtz, D. M.2018	1	0.5	1	1	0.5	1	1	6
Sarkozy, C.2017	1	1	1	1	0.5	1	1	6.5
Fontanilles, M. 2017	1	1	1	1	1	1	1	7
Hossain, N. M.2019	0.5	1	0.5	1	0.5	1	1	5.5
Qiong Li.2020	1	1	1	1	1	1	1	7

### Statistical Analysis

Diagnostic variables, such as sensitivity, specificity, likelihood ratios [i.e., positive likelihood ratios (PLR) or negative likelihood ratios (NLR)], diagnostic ratios (DOR), and the summary receiver operating characteristic curve (SROC) were calculated and analyzed using the Meta-Disc software, version 1.4. The pooled HR and the 95% CIs for OS or PFS were analyzed by the Review Manager version 5.4 software. We used a random-effect model if significant heterogeneity was observed (*P* < 0.05 or *I*^2^ > 50%); if not, we would turn to a fixed-effect model.

## Results

**Figure 1:**
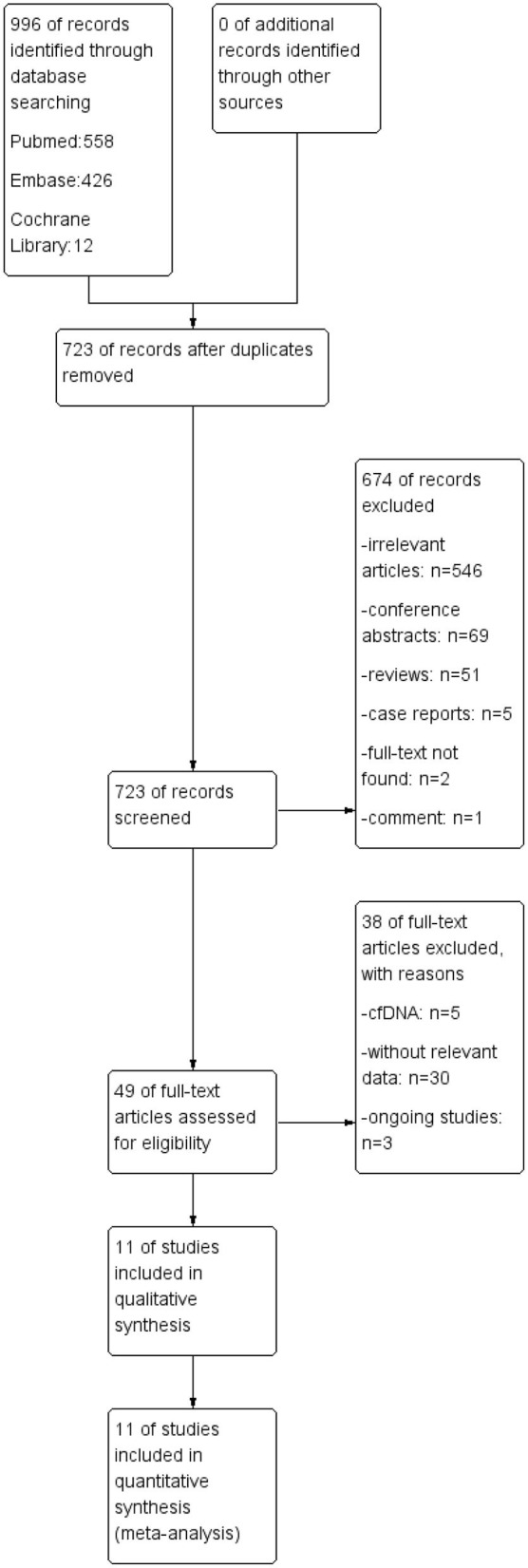
Flow chartof selection process to enrolleligiblestudie.

A total of 996 articles were identified through the search (Figure 1). After screening, 273 duplicated studies were removed, and 674 studies were excluded based on their titles and abstracts. A further 39 studies were excluded for not fulfilling the inclusion criteria. Finally, a total of 11 studies were included in the meta-analysis. The details and main characteristics of included studies are summarized in Table 4.

**Table 4:** Major characteristics of enrolled studies.

**No**.	**Study**	**Type of cancer**	**Number of patients**	**ctDNA measurement method**	**Mutation evaluated in ctDNA**
1	Fontanilles, M. (14)	PCNSL	50	NGS	MYD88 c.T778C
2	Hickmann, A, K. (15)	PCNSL	6	NGS	MYD88, PIM1, BCL2, ETV6, KMT2D, LPHN3, PRDM1, CD79B, SOCS1, IRF4, MYC, FOXB1, LRP1B, HLA-B, LTF, EPHA5, EP400, SYNE1, BLNK, STAT3, FES, SEPT9, POLR3A, DPYD, TFE3, CSMD3, FAT1, KIT, MAGI1, TP53, ASXL1, SETD2, IDH2, MTRR, PBRM1, BCR, MN1, RNF213, TOP1, ATM, FANCM, CDKN1B, PAX5, FOXO1, MCL1, PTPRT, CARD11, PPM1D, DST, BCL10, TCL1A, FN1, HSP90AA1, NIN, SLCO1B1, HSP90AB1, ARID5B, ETS1, ERBB4, CCND2, HLA-C, ITGB2, EPHA3, BCL6, TBL1XR1, PCBP1, RECQL4, CREBBP, STAT4, MLLT3, KEAP1, BTK
3	Mazzotti,C. (16)	MM	37	NGS	IGH, IGK, IGL rearrangements
4	Sakata-Yanagimoto, M. (17)	PTCL	14	Targeted sequencing	G17V-RHOA
5	Watanabe, J. (18)	PCNSL	12	ddPCR	MYD88
6	Roschewski, M. (19)	DLBCL	126	NGS	VDJ rearrangements
7	Assouline, S. E. (20)	DLBCL	20	CAPP-seq and ddPCR	EZH2, MEF2B, CREBBP, EP300, MLL2, FAS, STAT6, TP53, MYD88, MLL3
8	Kurtz, D. M. (21)	DLBCL	217	CAPP-seq	ABCB11, ACTG1, AFF1, APC, B2M, BCHE, BCL10, BCL2, BCL6, BTG1, BTG3, BTK, CAPZA3, CARD11, CCND1, CCND3, DSEL, EGR1, EPHA7, RF1, IRF4, KRAS, LAMA1, MAP2K1, MC5R, MED12, MEF2B, PXDN, RASSF9, STAT6, ZFP42, ZMYM6, ZNF608, ZNF678, et al.
9	Sarkozy, C. (22)	FL	29	NGS	VDJ rearrangements
10	Hossain, N. M. (23)	DLBCL	6	NGS	immunoglobulin gene V(D)J rearrangements
11	Qiong, Li. (24)	ENTKL	65	NGS	ADAM3A, APC, ARID1A, ARID1B, ARID2, ASXL3, ATM, BCOR, BCORL1, CD28, CHD8, CREBBP, DDX3X, DNMT3A, EP300, EZH2, FYN, IDH2, IL2RG, JAK1, JAK3, KDM6A, KMT2A, KMT2D, MGA, NF1, NOTCH1, PRDM1, PTPN1, RHOA, SETD2, SOCS1, STAT3, STAT5B, STAT6, TET1, TET2, TNFRSF14, TP53, TRAF3, ZAP608

### ctDNA as Marker for Diagnosis in Hematological Malignancies

**Figure 2:**
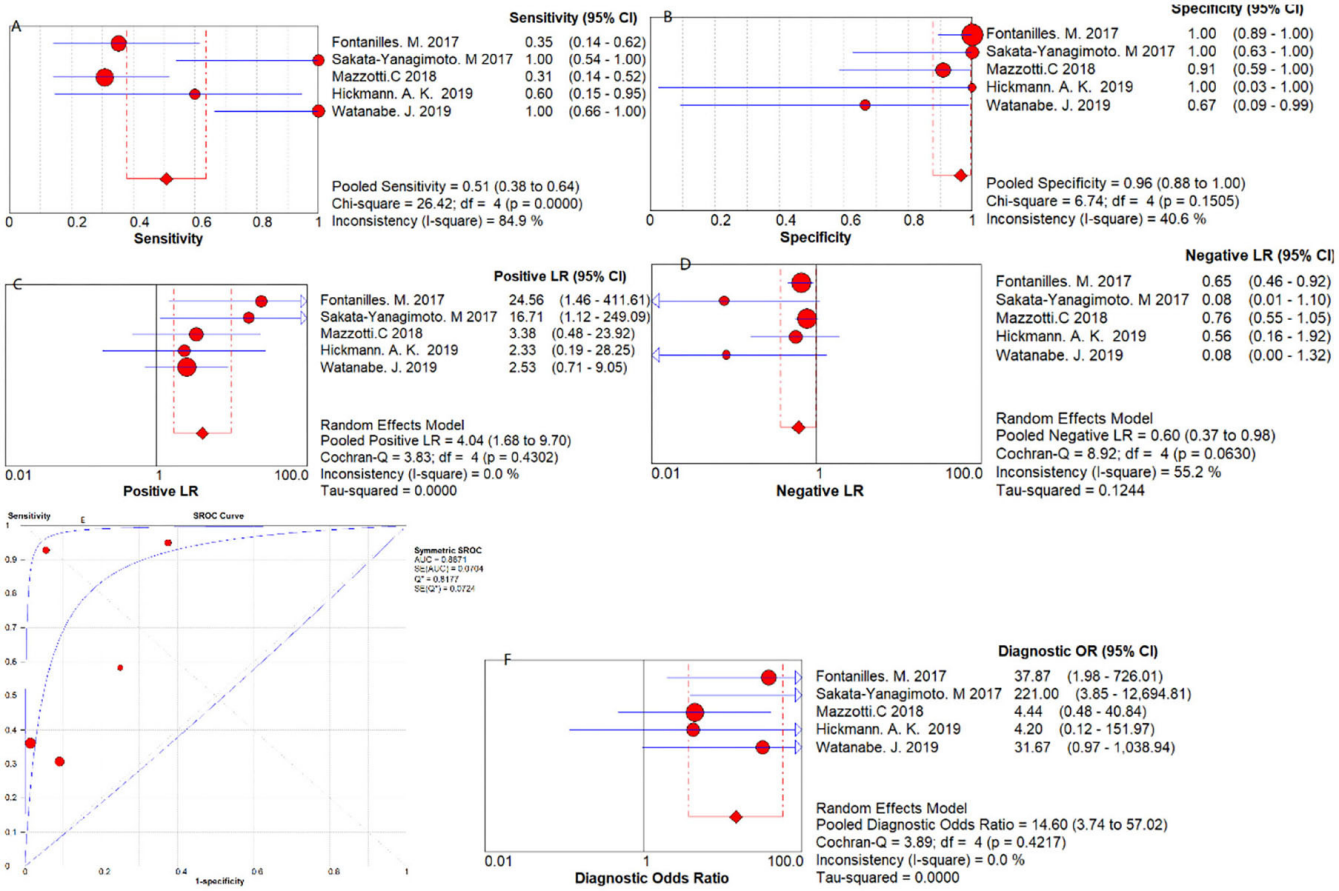
Diagnostic accuracy forest plots. **(A)** Forest plots of overall sensitivity. **(B)** Forest plots of overall specificity. **(C)** Forest plots of positive likelihood ratio. **(D)** Forest plots of negative likelihood ratio. **(E)** Forest plots of SROC Curve. **(F)** Forest plots of diagnostic odds ratio.

Five studies were pooled for the meta-analysis of diagnostic accuracy. As presented in Figure 2, the overall sensitivity and specificity was 0.51 (95% CI 0.38–0.64) and 0.96 (95% CI 0.88–1.00) respectively. The pooled PLR and NLR were 4.04 (95% CI 1.68–9.70) and 0.60 (95% CI 0.37–0.98), respectively. The area under the SROC was 0.89 and the DOR was 14.60 (95%CI 3.74–57.02).

### ctDNA as Prognostic Marker in Hematological Malignancies

**Figure 3:**
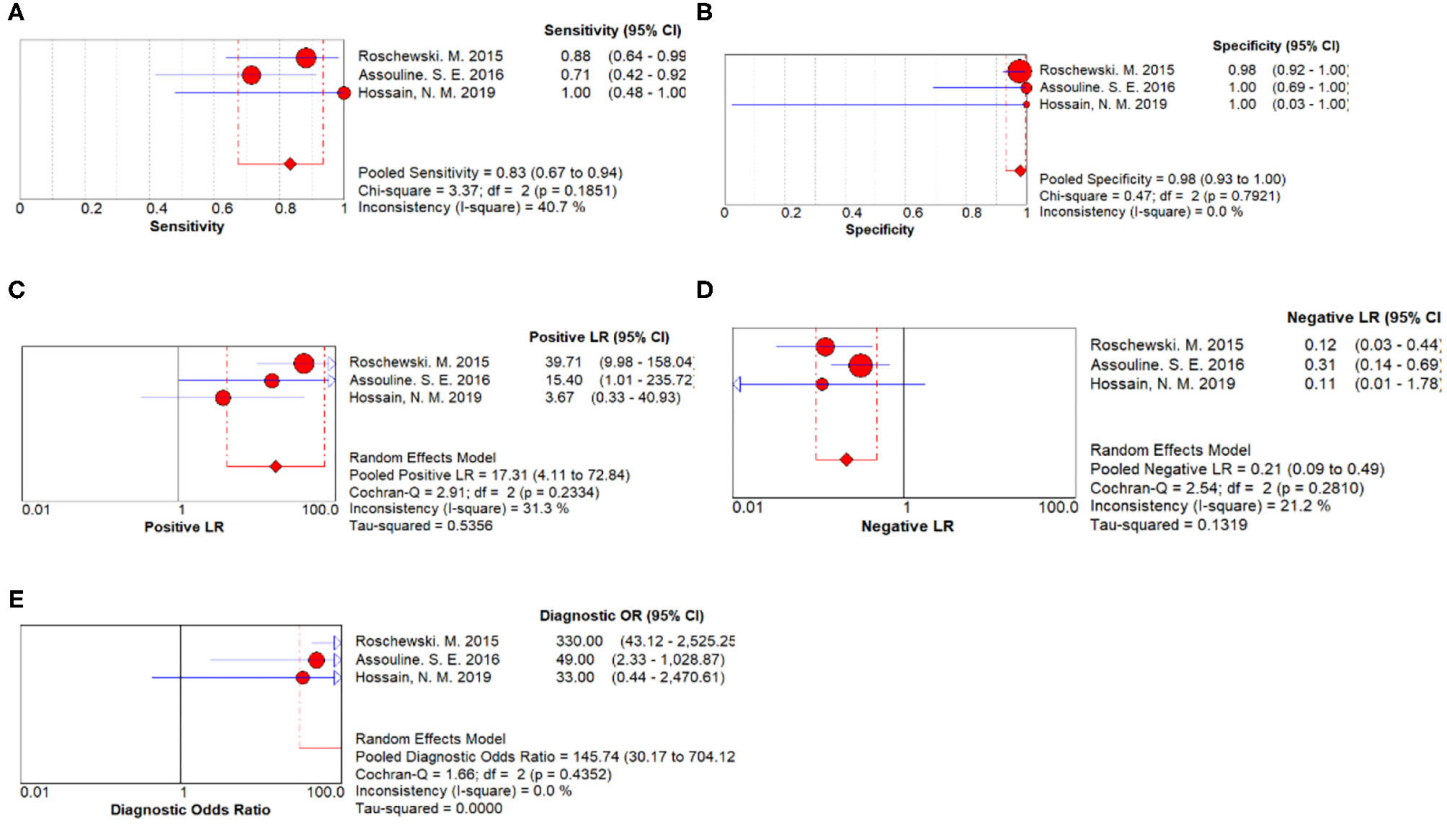
Disease progression prediction forest plots. **(A)** Forest plots of overall sensitivity. **(B)** Forest plots of overall specificity. **(C)** Forest plots of positive likelihood ratio. **(D)** Forest plots of negative likelihood ratio. **(E)** Forest plots of diagnostic odds ratio.

Three studies were pooled for the meta-analysis of disease progression prediction. As shown in Figure 3, the overall sensitivity and specificity was 0.83 (95% CI 0.67–0.94) and 0.98 (95% CI 0.93–1.00) respectively. The pooled PLR and NLR were 17.31 (95%CI 4.11–72.84) and 0.21 (95% CI 0.09–0.49), respectively. The DOR was 145.74 (95% CI 30.17–704.12).

**Figure 4:**
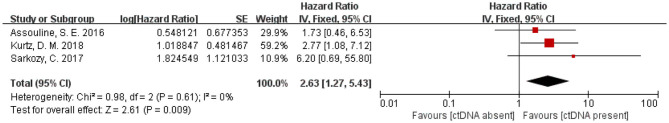
Forest plot of a Fixed-effect meta-analysis of the prognostic role of ctDNA on progression-free survival.

**Figure 5:**
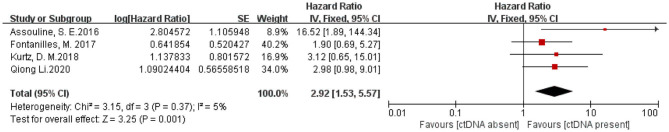
Forest plot of a Fixed-effect meta-analysis of the prognostic role of ctDNA on overall survival.

Three articles with a total of 266 patients included analysis on the association of ctDNA and PFS in patients with hematological malignancies. A significantly worse PFS for ctDNA positive patients was observed(HR 2.63, 95% CI 1.27–5.43)(Figure 4). Moreover, there were four articles with 352 patients that included analysis on the association of ctDNA and OS with hematological malignancies. Patients who had ctDNA positive or higher ctDNA levels had a worse OS (HR 2.92, 95% CI 1.53–5.57) (Figure 5).

## Discussion

Since fragmented DNA was first found in the whole blood by Mandel and Metais, cfDNA and ctDNA have been applied in a variety of disciplines. For example, detection of ctDNA is an effective method to determine EGFR status in NSCLC, providing a more expedient measure to predict resistance to EGFR tyrosine kinase inhibitors and prognosis (25). A study showed that ctDNA monitoring may help identify hematologic cancer patients at risk for relapse in advance of established clinical parameters (8). However, the relationship between ctDNA and hematological malignancies remains unclear. Therefore, it is necessary to conduct comprehensive analysis illuminating the clinical utility of ctDNA in the diagnoses of patients with hematological malignancies and prognosis prediction.

Our pooled data have shown that the detection of ctDNA has an obvious advantage in hematological malignancies diagnosis specificity (specificity: 0.96, 95% CI 0.88–1.00). A phase 1 clinical trial which studied the clinical value of ctDNA in MDS showed that there was an excellent correlation (*r*^2^ = 0.84; *P* < 0.0001) between the mutant allele fraction (MAF) of somatic mutations in BM and ctDNA across multiple matched time points (26). Another study on gene detection with 26 patients with MDS showed that the correlation of 52 somatic mutations detected in BM and ctDNA was also significant (*R*^2^ =0.8272, *P* < 0.0001) (13). These results imply that mutations in ctDNA may represent somatic mutations in tumor cells. So in this regard, ctDNA testing is a good alternative to biopsy because of its non-invasive advantages. However, in terms of test sensitivity, the present evidence showed no superiority of ctDNA over biopsy (sensitivity: 0.51, 95% CI 0.38–0.64). Hematologic tumors are highly heterogeneous, with various gene mutations in a tumor, which may be one of the main reasons for its lack of sensitivity. On the other hand, we pooled results of various tumors and mutations on account of the limited number of studies in our meta-analysis, which may lead to inaccurate results. Therefore, more accurate circulating gene targets need to be defined. To increase the sensitivity of ctDNA, there is a necessity to study the detection rate of ctDNA using a panel-based NGS approach, and panels should include the most-frequently mutated genes in tumor tissue.

The quantitative level of ctDNA has prognostic value for patients which could influence therapy choices (27, 28). Detection of ctDNA clearance during first-line chemotherapy can reflect tumor response to treatment, which may allow real-time adjustment of duration or intensity, or identification of patients at high risk of treatment failure (20). Similar to our results, studies by Herrera et al. also showed a correlation between ctDNA and disease progression as well as recurrence, whose multivariable model results show that detectable ctDNA was associated with increased risk of progression/death (HR 3.9, *P* = 0.003) and relapse/progression (HR 10.8, *P* = 0.0006) (29). Moreover, some studies indicated that detectable ctDNA was also associated with tumor volume, which means ctDNA is associated with tumor burden in patients (11, 24). Given the non-invasive nature and short half-life of ctDNA, its interim monitoring during therapy can provide a real-time assessment of tumor dynamics, allowing for an early indication of response or resistance to therapy. After treatment, ctDNA can be used for post-treatment monitoring. It can perform a similar function to surveillance imaging without the need for radiation exposure, which may potentially increase susceptibility to preclinical recurrence, and ultimately allow for early intervention (28, 30, 31). The study by Roschewski et.al. showed that patients developed detectable ctDNA a median of 3.5 months before clinical evidence of disease (20). Similarly, ctDNA detected relapse at a mean of 6 months before imaging detection in another study by Scherer et al. (32). These encouraging results suggest that ctDNA may help faster relapse detection, and allows subsequent therapy to be initiated before clinical progression. These findings will highly strengthen the value of ctDNA in clinical management of patients with hematological malignancies.

As is shown in our pooled data, the presence of ctDNA or higher levels of ctDNA is associated with a poorer PFS and OS. Sarkozy's study has a similar result to us, showing that patients with higher levels of ctDNA experienced a significantly shorter PFS than those with lower levels of ctDNA (median 15.3 months vs. not reached, *p* = 0.004) (23). Another study also showed that patients with detectable ctDNA have worse survival outcomes than those without (6). These results imply that ctDNA assessment could be a useful alternative endpoint for PFS and OS.

Several limitations in this study need to be addressed. Firstly, the lack of a well-accepted ctDNA gene target might contribute to the presence of bias. Secondly, due to the limited studies, we included little data which may lead to results' bias. Furthermore, the difference in detection method and materials, such as PCR primers or the equipment applied, is also an important source of study bias. Therefore, our conclusion might not be universal suitable.

Despite its preliminary nature, this study clearly indicated that the presence of ctDNA in hematological malignancies patients predicted unfavorable survival. Before its wide application in hematological malignancies patients, some concerns still need to be addressed, including more accurate molecule targets and more suitable detection techniques. In a word, more prospective studies with consistent and standardized methodology are needed to further resolve these problems.

## Conclusions

In summary, our meta-analysis revealed that the presence of ctDNA is related to a worse prognosis in patients with hematological malignancies (lymphomas, multiple myeloma, myelodysplastic syndrome, or leukemia). Moreover, ctDNA is a potential diagnostic biomarker in hematological malignancies, although the low diagnostic accuracy is a point of concern. The specificity and non-invasive nature of ctDNA testing, as well as its ability to reflect the patient's tumor burden in real time, makes it a potential substitute for biopsy. In the future, more studies are needed to realize the standardization of sequencing techniques and explore methods to improve detection sensitivity.

## Data Availability Statement

The original contributions presented in the study are included in the article/Supplementary Material, further inquiries can be directed to the corresponding author/s.

## Author Contributions

XYT and HY collected and analyzed the data and wrote the paper. LC analyzed the data. YYZ revised the paper. CYS conceived and designed this study, analyzed the data, and wrote the paper. All authors read, reviewed, and approved the final manuscript.

## Conflict of Interest

The authors declare that the research was conducted in the absence of any commercial or financial relationships that could be construed as a potential conflict of interest.

## Abbreviations

ctDNA: circulating tumor DNA
BM: bone marrow
cfDNA: cell-free DNA
ddPCR: droplet digital polymerase chain reaction
NGS: next-generation sequencing
HR: hazard ration
OS: overall survival
PFS: progress-free survival
EFS: event-free survival
CI: confidence interval
PLR: positive likelihood ratios
NLR: negative likelihood ratios
DOR: diagnostic ratios
SROC: the summary receiver operating characteristic curve
EGFR: epidermal growth factor receptor
MRD: the minimal residual disease
MAF: the mutant allele fraction
MDS: myelodysplastic syndrome.
