# Supplementary material for: Clinical Value of ctDNA in Hematological Malignancies (Lymphomas, Multiple Myeloma, Myelodysplastic Syndrome, and Leukemia): A Meta-Analysis
Source: Front Oncol. 2021 Mar 4;11:632910. doi: 10.3389/fonc.2021.632910 (PMC7970179; doi:10.3389/fonc.2021.632910)
Supplement: Supplementary file 1 [file Table_1.docx]

Supplementary table1: raw data for picture 2

| study | TP | FP | FN | TN |
| --- | --- | --- | --- | --- |
| Fontanilles, M. 2017 | 6 | 0 | 11 | 33 |
| Sakata-Yanagimoto, M 2017 | 6 | 0 | 0 | 8 |
| Mazzotti.C 2018 | 8 | 1 | 18 | 10 |
| Hickmann, A. K. 2019 | 3 | 0 | 2 | 1 |
| Watanabe, J. 2019 | 9 | 1 | 0 | 2 |

Supplementary table1: raw data for picture 3

| study | TP | FP | FN | TN |
| --- | --- | --- | --- | --- |
| Roschewski, M.2015 | 15 | 2 | 2 | 88 |
| Assouline, S. E. 2016 | 10 | 0 | 4 | 11 |
| Hossain, N. M. 2019 | 5 | 0 | 0 | 1 |

TP: true positive; FP: false positive; FN: false negative; TN: true negative
